# A case report of multiple cervical artery dissection after peripheral type facial palsy and use of steroids

**DOI:** 10.1186/s12883-018-1080-x

**Published:** 2018-05-28

**Authors:** Sung eun Chung, Tae Hwan Yoon, Kyung Mi Lee, Hyug-Gi Kim, Bum Joon Kim

**Affiliations:** 1 Department of Neurology, Kyung Hee University Hospital at Gangdong, Seoul, Republic of Korea; 2 Department of Neurology, Kyung Hee University Hospital College of Medicine, Kyung Hee University, 23, Kyung Hee Dae-ro, Dongdaemun-gu, Seoul, 190 Republic of Korea; 3 Department Radiology, Kyung Hee University Hospital, Seoul, Republic of Korea

**Keywords:** Cervical artery dissection, Systemic steroid, Bell’s palsy

## Abstract

**Background:**

Cervical artery dissection is one of the most important causes of ischemic stroke in young age patients. However, multiple cervical artery dissection simultaneously involving the anterior and posterior circulation is uncommon. Here, we would like to report a case of a patient with bilateral vertebral artery (VA) and internal carotid artery dissection (ICA) after a use of systemic steroid due to peripheral facial palsy.

**Case presentation:**

A 44-year-old man with hypertension visited emergency department due to recurrent vertigo. He was receiving methyl prednisolone for two weeks for the treatment of right peripheral type facial palsy which occurred after retro-orbital headache. Neurologic examination revealed severe ataxia at left side. Sensory for pain and temperature was declined in the right arm and leg. Diffusion-weighted image showed an acute ischemic lesion at the whole territory of posterior-inferior cerebellar artery. Severe stenosis was observed from bilateral VAs and ICAs on conventional magnetic resonance angiography. Intramural hematoma and intimal flap was observed from the high-resolution MRI.

**Conclusions:**

Peripheral type facial palsy is an unusual presentation of carotid dissection. Steroids aggravate arterial dissection by increasing blood pressure and blood vessel fragility by its negative effect on connective tissue strength. Use of steroid in patients with peripheral type facial palsy with severe headache may need caution.

## Background

Arterial dissection is one of the most important causes of ischemic stroke in young age patients [1]. Dissection begins as a tear in one of the carotid arteries of the neck, which allows blood under arterial pressure to enter the wall of the artery and split its layers [2]. Cervical artery dissection (CeAD) causes ischemic stroke, Horner syndrome and/or multiple cranial nerve palsies, usually involving the lower cranial nerves [3]. However, there has been several reports of upper cranial nerve palsy resulting from spontaneous internal carotid artery (ICA) dissections [4].

The reason of spontaneous CeAD is not clarified in most cases. Only half of arterial dissection is associated with a trauma history, and more less are associated with overt connective tissue disease [5]. Therefore other unknown systemic factors may affect arterial dissections [6]. Several drugs are known to influence the risk of arterial dissections [7]. Here, we would like to report a case of a patient with multiple CeAD involving bilateral distal ICA and vertebral artery (VA) after use of steroid due to peripheral type facial palsy.

## Case presentation

A 44-year-old man with hypertension visited emergency department (ED) due to recurrent vertigo. Two weeks before admission, he visited the ED with a sudden onset severe headache in the retro-orbital area and right peripheral type facial palsy of House-Brackmann scale grade 4. No skin lesion was observed from the periauricular area and the external auditory canal. Computerized tomography was unremarkable. He was diagnosed as Bell’s palsy and received methylprednisolone 60 mg once daily for one week and then was under a scheduled tapering. He had history of hypertension and was under regular medication (amlodipine 5 mg and fimasartan 60 mg, once a day) for the last 2 years.

Thirteen days later, the patient felt headache aggravation after stretching himself and just after a few seconds the vertigo began. At the second visit to the ED, initial blood pressure was 184/105 mmHg. Neurologic examination revealed severe ataxia at left side. Sensory for pain and temperature was declined in the right arm and leg. Horner syndrome including miosis and ptosis of the left eye was observed. There also was gaze evoked nystagmus during left lateral gaze. The peripheral type right facial palsy remained. There was no family history, symptom or sign of any connective tissue disorder or a history of external trauma. There were no findings of secondary hypertension from an extensive evaluation.

**Fig. 1:**
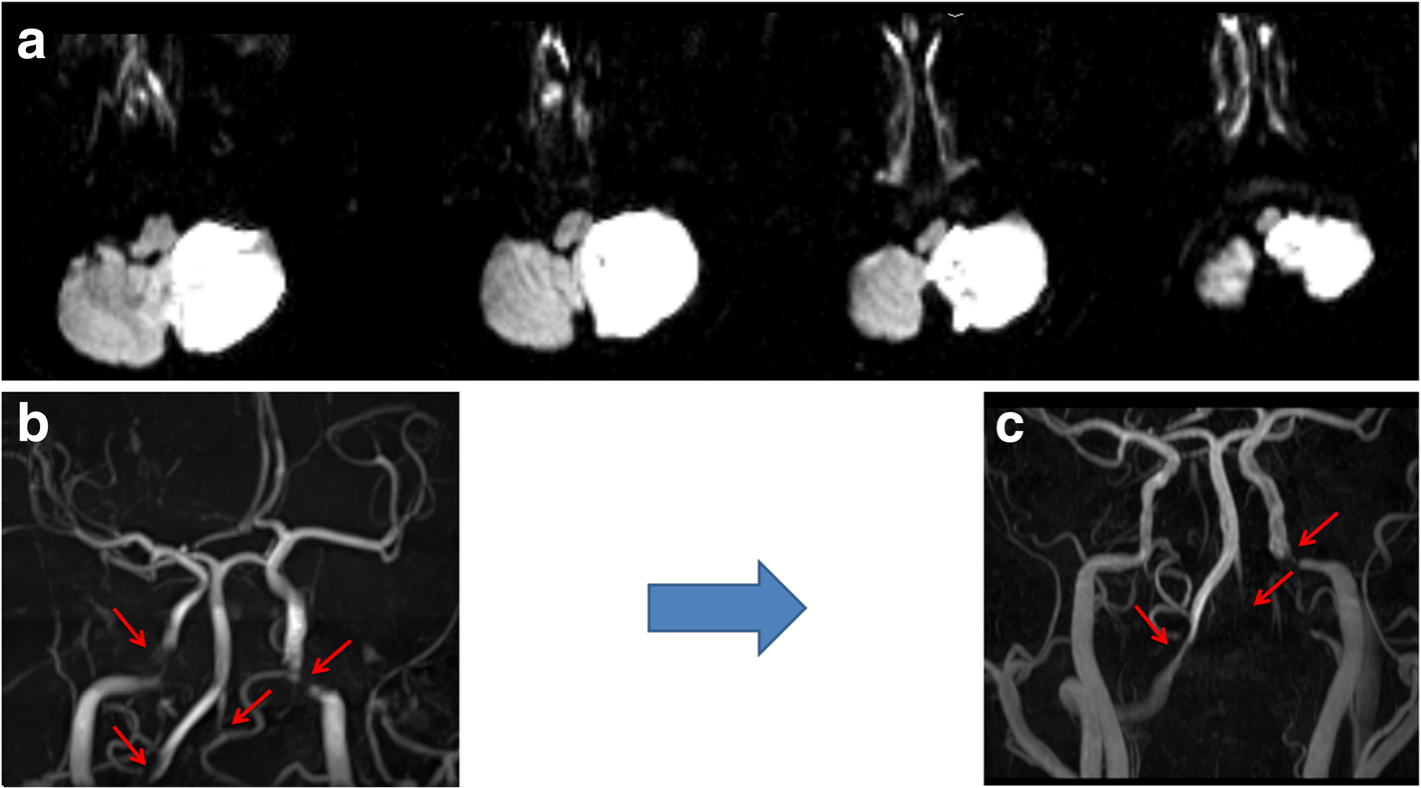
Diffusion weighted image (**a**) and time-of-flight magnetic resonance image initial (**b**) and at follow-up (**c**)

**Fig. 2:**
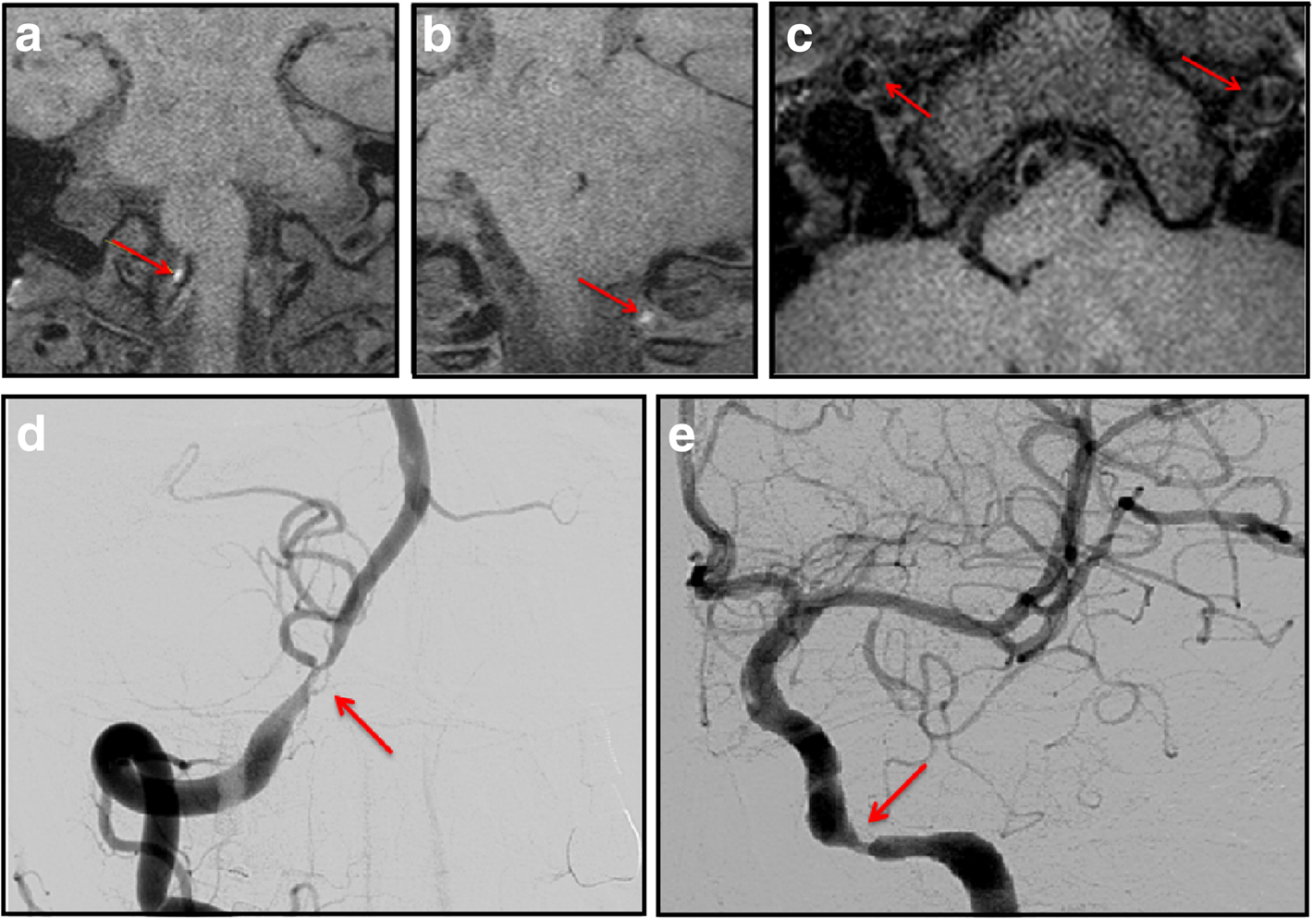
High-resolution MRI showing intramural hematoma at right (**a**; coronal image) and left vertebral arteries (**b**; coronal image) and dissecting flap in bilateral distal internal carotid arteries (**c**; axial image) Digital subtraction angiography shows severe stenosis at right vertebral artery (**d**) and left distal internal carotid artery (**e**)

Magnetic resonance image (MRI) was taken from ED; an acute ischemic lesion was observed at the whole territory of left posterior inferior cerebellar artery at cerebellum with an additional lesion at left lateral medulla from diffusion weighted image (Fig. 1a). From the time-of-flight MR angiography (TOF-MRA), severe stenosis was observed from bilateral VAs and ICAs (Fig. 1b). TOF-MRA with high-resolution MR was followed up 2 days later; the stenosis at right distal ICA was improved (Fig. 1c) and, an intramural hematoma was observed from bilateral VA (Fig. 2a and b) and an intimal flap was observed from bilateral distal ICAs (Fig. 2c). Digital subtraction angiography was performed; left VA was occluded. Right VA and left ICA demonstrated severe stenosis (Fig. 2d and e). However, as dissection was multiple, located at eloquent area and not clinically worsening after the first stroke, intervention was not considered initially and antiplatelet agent was used. The patient was followed up 2 months after the initial event. There was no further ischemic event and the patient recovered to have mild ataxia and sensory change. The peripheral type facial palsy improved to a satisfactory level (House-Brackmann scale grade 2). Informed consent was obtained from the patient including the consent for the publication of all the personal, medical details and images.

## Discussion and conclusion

We described a patient with extensive dissection at four cervical arteries, which includes bilateral VA and ICAs after use of steroid to treat peripheral type facial palsy. Multiple CeADs are observed from ~ 15% of all CeADs. Mostly, multiple CeAD involved two vessels, whereas ~ 1% involved three cervical arteries, and only 0.1% demonstrated quadruple CeAD [5, 8].

It is well-known that a systemic factor – infection - may precede multiple CeAD [8, 9]. Interestingly, a history of preceding infection was associated with multiple CeADs, whereas a history of recent trauma, which may act more locally, was not [10]. Our patient had a history of peripheral type facial palsy, which is also considered to be triggered by viral infections [11]. But, it is not clearly verified whether there is an association between peripheral type facial palsy and CeAD. In the other hand, the initial left facial palsy may have been associated with the left distal ICA dissection which may have been occurred before the ischemic event, rather than by Bell’s palsy. There were several case reports reporting ipsilateral peripheral type facial palsy due to distal ICA dissection, explained by disruption of anomalous nutrient artery and/or nerve ischemia due to a transient or permanent interruption of the blood supply by compression of the vasa nervorum originating from the intracranial carotid artery [12–14]. This vascular theory, explaining facial palsy in those with ICA dissection by ischemia, may be supported by several reports of facial palsy occurring during intra-arterial procedures [15].

Our patient also received steroid for two weeks before dissection. Several reports demonstrate that even a short use of steroid may trigger arterial dissections of aorta or coronary arteries [16, 17]. Steroids increase blood pressure and increases blood vessel fragility by its negative effect on collagen formation and connective tissue strength [18]. Previously a patient with VA dissection after the use of anabolic steroid was reported, but was not a case of multiple CeAD [19]. There may be a chance that a preceding left ICA dissection caused peripheral type facial palsy, and the use of steroid with uncontrolled hypertension may have aggravated the dissection into multiple CeADs.

In our case, antiplatelet agent was used to prevent further ischemic event. Because the dissections were located intracranial, extension of dissections to the adventitia may cause subarachnoid hemorrhage. From a previous study comparing antiplatelet agent and anticoagulation, there was no difference in terms of efficacy [20]. Stenting or angioplasty can be considered when disruption of flow is observed, but as the dissection occurred in multiple sites, at an eloquent area and without clinical worsening or fluctuations, medical treatment was preferred [21].

The first two weeks after dissection shows the highest risk of stroke [22]. Therefore, early diagnosis and symptom detection of dissection may be critical. Peripheral type facial palsy may be an unusual presentation of ICA dissection. It is unclear whether the peripheral type facial palsy was induced by Bell’s palsy due to viral infection or dissection in our patient. However, considering that steroid can induce or aggravate arterial dissection, use of steroid in patients with peripheral type facial palsy, especially in those with retro-orbital headache may need caution with detailed vascular imaging to rule out ICA dissection.

## Abbreviation

CeAD: Cervical artery dissection
ED: Emergency department
ICA: Internal carotid artery
MRI: Magnetic resonance image
TOF-MRA: Time-of-flight magnetic resonance angiography
VA: Vertebral artery
